# A Comparative Study on the Cost of New Antibiotics and Drugs of Other Therapeutic Categories

**DOI:** 10.1371/journal.pone.0000011

**Published:** 2006-12-20

**Authors:** Matthew E. Falagas, Konstantinos N. Fragoulis, Ioannis Karydis

**Affiliations:** 1 Alfa Institute of Biomedical Sciences Athens, Greece; 2 Department of Medicine, “Henry Dunant” Hospital Athens, Greece; 3 Department of Medicine, Tufts University School of Medicine, Boston Massachusetts, United States of America; James Cook University, Australia

## Abstract

**Background:**

Drug treatment is becoming more expensive due to the increased cost for the introduction of new drugs, and there seems to be an uneven distribution of medication cost for different therapeutic categories. We hypothesized that the cost of new antimicrobial agents may differ from that of other therapeutic categories and this may play a role in the stagnation of development of new antibiotics.

**Methodology/Principal Findings:**

We performed a pharmaco-economical comparative analysis of the drug cost of treatment for new agents introduced in the United States drug market in various therapeutic categories. We calculated the drug cost (in US dollars) of a ten-day treatment of all new drugs approved by the FDA during the period between January 1997 and July 2003, according to the 2004 Red Book Pharmacy's Fundamental Reference. New anti-neoplastic agents were found to be the most expensive drugs in comparison to all other therapeutic categories, with a median ten-day drug-treatment cost of US$848 compared to the median ten-day drug-treatment costs of all other categories ranging from US$29 to US$301. On the other hand, new antimicrobial drugs were found to be much less expensive, with a median ten-day drug-treatment cost of US$137 and $US85 for all anti-microbial agents and for anti-microbial agents excluding anti-HIV medications, respectively.

**Conclusions/Significance:**

The drug-treatment cost of new medications varies considerably by different therapeutic categories. This fact may influence industry decisions regarding the development of new drugs and may play a role in the shortage of new antimicrobial agents in the fight against the serious problem of antimicrobial resistance.

## Introduction

The investment of societies around the world on biomedical research leads to important developments including the continuous discovery of new pharmaceutical compounds. It is not clear, however, whether newly developed and approved drugs cover all therapeutic areas in a relatively uniform manner. Different reasons might be postulated with respect to the driving forces of drug development [1].

Some may argue that more drugs are being developed in areas where the benefits to the society are maximized whereas others may claim that the direction is led to where the economic incentive for the pharmaceutical industry is higher [2]–[5]. A third reason may be a mounting social pressure from rising prevalence and incidence of certain health problems. Other reasons, which may influence drug development, may include the failure of current medications to solve existing health problems or the identification of new diseases such as the highlighted example of AIDS. The problem of new drugs–usually more expensive - substituting for older ones with similar chemical structure, which are loosing patent privileges, is thought to be one of the factors contributing to the increasing annual drug expenditure around the world.

The problem of shortage of new anti-microbial agents has been well described in the medical literature [6]. This disturbing fact is occurring despite the gradually increasing prevalence of microbial resistance to existing antibiotics in various parts of the word [7]–[9]. A significant reduction (56%) of new approvals of antibacterial agents occurred during the period 1998–2002 compared to the period 1983–1987. In addition, the fact that only 6 out of 506 drugs disclosed in the development programs of the largest pharmaceutical and biotechnology companies are antibacterial agents is really distressing [10].

We hypothesized that the cost of new antimicrobial agents compared to new medications in other therapeutic areas may be a reason that plays a significant role in the relatively stagnated market of new anti-microbial agents, especially for non-HIV infectious diseases. Thus, we performed a pharmaco-economical study in order to analyze the cost of newly approved drugs and compare the overall treatment cost for new drugs between different therapeutic categories.

## Methods

For the purpose of our study we analyzed data regarding original new drugs applications approved by the U.S. Food and Drug Administration (FDA) from January 1997 to July 2003. The data were obtained from the FDA website [11]. Based on the FDA definition of original new drugs, compounds that have the same chemical structure with an already approved medication are not considered new drugs.

In order to conduct our analysis, we classified each new drug in one of sixteen main therapeutic categories. However, four therapeutic categories were excluded from further analysis because of the small number of new drugs developed in each category. The excluded categories were: contrast agents [including 3 new drugs (Tc-99m depreotide, Tc-99m apcitide, and human albumin microspheres)], antidotes [including 1 new drug (fomepizole)], ear, nose and throat drugs [including 1 new drug (cevimeline)], and anesthesiology drugs (there was no new medication in this category during the study period). In addition, urea C-14 was not included in the gastrointestinal category since it is used exclusively for diagnostic purposes.

The cost of each new drug was determined using the Average Wholesale Price found in the Red Book Pharmacy's Fundamental Reference, 2004 edition [12]. In order to compare the treatment cost of new drugs for different therapeutic categories, we made several assumptions. First, we calculated the cost of a ten-day drug-treatment for each new drug. For drugs that are used for a shorter period than ten days, we defined as “ten-day drug-treatment cost”, the price of the drug formulation with the fewer possible units of the medication required for each patient. We then calculated the median, mean, and the range of the ten-day drug-treatment cost of new drugs in the defined therapeutic categories.

## Results

One hundred and twenty-nine new drugs were approved by the FDA during the study period (January 1997 to July 2003). In Table 1 we present 124 new drugs approved, classified in 12 therapeutic categories that we analyzed further. In Table 2 we present the number of new drugs by therapeutic category as well as summary data on the drug cost of a ten-day treatment. All drug prices were presented in 2004 U.S. dollars (USD).

**Table 1:**
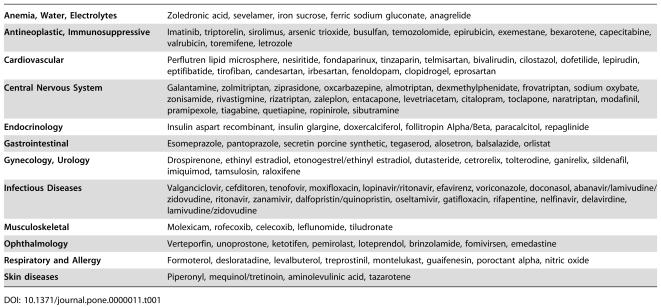
Original new drugs in different therapeutic categories by chronological order of approval from the FDA (January 1997–July 2003).

**Anemia, Water, Electrolytes**	Zoledronic acid, sevelamer, iron sucrose, ferric sodium gluconate, anagrelide
**Antineoplastic, Immunosuppressive**	Imatinib, triptorelin, sirolimus, arsenic trioxide, busulfan, temozolomide, epirubicin, exemestane, bexarotene, capecitabine, valrubicin, toremifene, letrozole
**Cardiovascular**	Perflutren lipid microsphere, nesiritide, fondaparinux, tinzaparin, telmisartan, bivalirudin, cilostazol, dofetilide, lepirudin, eptifibatide, tirofiban, candesartan, irbesartan, fenoldopam, clopidrogel, eprosartan
**Central Nervous System**	Galantamine, zolmitriptan, ziprasidone, oxcarbazepine, almotriptan, dexmethylphenidate, frovatriptan, sodium oxybate, zonisamide, rivastigmine, rizatriptan, zaleplon, entacapone, levetriacetam, citalopram, toclapone, naratriptan, modafinil, pramipexole, tiagabine, quetiapine, ropinirole, sibutramine
**Endocrinology**	Insulin aspart recombinant, insulin glargine, doxercalciferol, follitropin Alpha/Beta, paracalcitol, repaglinide
**Gastrointestinal**	Esomeprazole, pantoprazole, secretin porcine synthetic, tegaserod, alosetron, balsalazide, orlistat
**Gynecology, Urology**	Drospirenone, ethinyl estradiol, etonogestrel/ethinyl estradiol, dutasteride, cetrorelix, tolterodine, ganirelix, sildenafil, imiquimod, tamsulosin, raloxifene
**Infectious Diseases**	Valganciclovir, cefditoren, tenofovir, moxifloxacin, lopinavir/ritonavir, efavirenz, voriconazole, doconasol, abanavir/lamivudine/zidovudine, ritonavir, zanamivir, dalfopristin/quinopristin, oseltamivir, gatifloxacin, rifapentine, nelfinavir, delavirdine, lamivudine/zidovudine
**Musculoskeletal**	Molexicam, rofecoxib, celecoxib, leflunomide, tiludronate
**Ophthalmology**	Verteporfin, unoprostone, ketotifen, pemirolast, loteprendol, brinzolamide, fomivirsen, emedastine
**Respiratory and Allergy**	Formoterol, desloratadine, levalbuterol, treprostinil, montelukast, guaifenesin, poroctant alpha, nitric oxide
**Skin diseases**	Piperonyl, mequinol/tretinoin, aminolevulinic acid, tazarotene

**Table 2:**
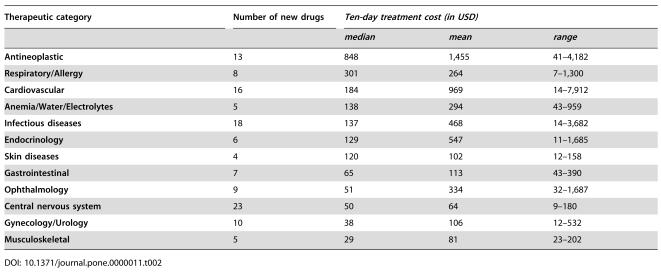
Number of original new drugs and ten-day treatment cost of different therapeutic categories (in USD - 2004).

Therapeutic category	Number of new drugs	*Ten-day treatment cost (in USD)*		
		*median*	*mean*	*range*
**Antineoplastic**	13	848	1,455	41–4,182
**Respiratory/Allergy**	8	301	264	7–1,300
**Cardiovascular**	16	184	969	14–7,912
**Anemia/Water/Electrolytes**	5	138	294	43–959
**Infectious diseases**	18	137	468	14–3,682
**Endocrinology**	6	129	547	11–1,685
**Skin diseases**	4	120	102	12–158
**Gastrointestinal**	7	65	113	43–390
**Ophthalmology**	9	51	334	32–1,687
**Central nervous system**	23	50	64	9–180
**Gynecology/Urology**	10	38	106	12–532
**Musculoskeletal**	5	29	81	23–202

The categories with the most expensive new medications were the antineoplastic and immunosuppressive agents, the drugs for the respiratory tract, and the cardiovascular medications. The median, mean, and range of the drug cost of a ten-day treatment with new anti-neoplastic agents were 848, 1,455, and 41–4,182 USD respectively. The corresponding numbers for the drug cost of treatment with new agents involving the respiratory system were 301, 264 and 7–1300 USD, for cardiovascular new agents 184, 969 and 14–7,912 USD, and for new anti-infectious agents 137, 468 and 14–3,682 USD. The median, mean, and range of the drug cost of a ten-day treatment with new agents used for the management of the HIV infection were 178, 302, and 105–1,080 USD respectively; the corresponding numbers for non-HIV antimicrobial agents were 85, 600, and 14–3,682 USD.

## Discussion

We studied the drug cost of the treatment of new agents to examine whether there are any important differences between the various therapeutic categories. It is interesting that the cost of treatment of new antineoplastic and immunosuppressive drugs is, by far, the highest compared to all other therapeutic categories. The drug-treatment cost of agents from the respiratory system and cardiovascular system category followed the anti-neoplastic agents category. The high drug cost, combined with the long duration of diseases for which most of these agents are prescribed, may explain the huge sales revenues from these drugs.

The median drug cost of a ten-day treatment of an antineoplastic agent was actually more than 6 times higher than the respective value in the category of antimicrobial agents; the difference is even more pronounced if someone excludes from the category of new antimicrobials, the drugs for the management of HIV infection. In fact, anti-neoplastic agents are given in prolonged cycles of chemotherapy and it is, therefore, reasonable to assume that our analysis actually underestimated the true drug cost of treatment for anti-neoplastic agents.

Although, we did not make an attempt to study the differences in the cost of pre-marketing development of new drugs in different therapeutic categories, we postulate that the observed differences in drug-treatment cost, and subsequently the drug sales revenue, may play a role in the decision making process for the introduction of new drugs. It may also be one of the factors that has led to a relative shortage of new antibiotics. It should be emphasized that the pre-marketing cost of development of new chemical entities has been found to be higher for anti-infective agents compared to several other categories of drugs, including cardiovascular and neuro-pharmacological agents [1].

Our study has several limitations. First, we had to measure the drug cost of a defined period of treatment (ten-day treatment) in order to provide some comparative estimates of treatment cost with new drugs in different therapeutic categories. The selection of any time period (e.g. one-day, one-week, one-month, etc) for our comparative analysis would have its own advantages and disadvantages given that the duration of treatment differs considerably for the numerous acute, sub-acute, and chronic diseases. Second, we calculated only the drug-treatment cost without taking into consideration other important factors that influence the cost of medical care, including cost related to the administration of parenteral medications. Third, we did not take into account data about the effectiveness and safety of new drugs in order to perform a comparative assessment of the cost-effectiveness of new agents in different therapeutic categories.

In addition, we did not examine the reasons for the considerable differences in the drug-treatment cost of with newly approved agents. Several factors may contribute to the observed differences in the cost; including the cost of development, production, and introduction of new drugs into the clinical practice [13]. Specifically, the cost of the infrastructure necessary for the development of new drugs may differ for different therapeutic categories. Furthermore, the cost of clinical trials, required for the approval of new drugs, may be also be different for the various therapeutic categories [14], [15]. For example, a longer follow up period of observation is necessary for the determination of the outcome in clinical trials studying anti-neoplastic compared to antibacterial agents. Moreover, differences in the failure rate of new drugs to successfully complete phases I to IV of clinical trials, may contribute to the increased cost of production of new anti-neoplastic and immunosuppressive drugs.

In conclusion, we found considerable differences in the drug-treatment cost of new medications between the various therapeutic categories. It is interesting to note that new drugs used for the treatment of infections are less expensive, especially if one excludes new agents for the management of HIV infection. It should be appreciated that the number of new antimicrobial drugs is actually quite high despite relatively low ten-day cost. However, this high number is obviously not sufficient in light of the prevalence/incidence of infectious diseases and the antimicrobial resistance. Additional comparative pharmaco-economical studies are needed to address the question whether new drugs provide good value for their cost. There is also a need to further explore the differences in the cost of new agents and the role they play in the relative stagnation in the development of new antibiotics, in an era when they are urgently needed because of the pandemic of antimicrobial resistance.
